# Invasive pyogenic infection and infective endocarditis due to *Streptococcus anginosus*: A case report

**DOI:** 10.1097/MD.0000000000018156

**Published:** 2019-11-27

**Authors:** Chiao-Wen Cheng, Cheng-Min Feng, Chian Sem Chua

**Affiliations:** a Department of Transportation and Logistics Management, National ChiaoTung University, Taipei, Taiwan; b Physician, Gastroenterologist & Hepatologist, Western Medicine Division, Hospital Lam Wah Ee, Malaysia; c Department of Medicine, Penang Medical College, Penang, Malaysia.

**Keywords:** brain microabscess, infective endocarditis, mitral valve prolapse, renal embolization, *Streptococcus anginosus*

## Abstract

**Rationale:**

*Streptococcus anginosus* mostly colonizes the digestive and genitourinary system, including the oropharyngeal region. It commonly causes invasive pyogenic infection, but less likely causes infective endocarditis (IE).

**Patient concerns:**

An 18-year-old woman who had an underlying mitral valve prolapse without mitral regurgitation presented to our hospital with low-grade fever, left leg weakness, and left abdominal pain. She was diagnosed with brain infarction and microabscess as well as IE. The patient totally recovered after the 6-week course of intravenous antibiotics.

**Diagnosis:**

Brain magnetic resonance imaging revealed brain infarction and microabscess. Abdominal computed tomography revealed splenic and left renal infarction. Three sets of blood culture were positive for *S anginosus*. Transthoracic echocardiogram identified mitral valve prolapse with moderate eccentric mitral valve regurgitation, and a 0.3 × 0.6-cm vegetation was found on the left mitral valve. All of these results meet the modified Duke criteria.

**Interventions:**

The abdominal pain and left leg weakness were improving after 2 weeks of intravenous antibiotics treatment. No neurological sequelae were noted after completing the 6-week course of medical treatment.

**Outcomes:**

The patient was successfully treated and discharged after completing the 6-week intravenous antibiotics treatment.

**Lessons:**

IE should be considered in young patients with native valve disease who have prolonged fever. Though *S anginosus* commonly causes invasive pyogenic infection, patients with native valve disease should be checked for IE.

## 1 Introduction

The incidence of infective endocarditis (IE) in Asia is increasing.^[1]^*Streptococcus anginosus*, is less likely associated with IE.^[2–4]^ Most of the *S anginosus* are colonized in the oropharyngeal region, digestive system, and genitourinary system.^[5]^ The most prevalent underlying diseases associated with IE are intravenous drug addiction and valve heart disease. In this report, we report a rare case of previously healthy patient with an underlying mitral valve prolapse acquired a *S anginosus* infection and presented with invasive brain abscess and IE as well as splenic and left renal embolization.

## 2 Case presentation

**Figure 1:**
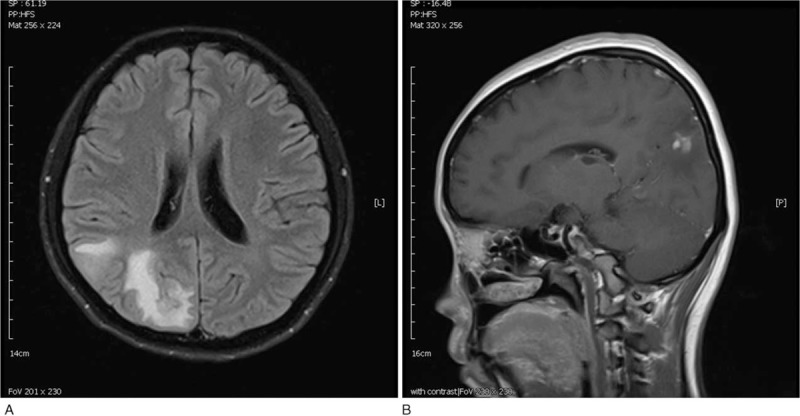
Brain magnetic resonance imaging showed disseminated septic emboli and microabscess.

**Figure 2:**
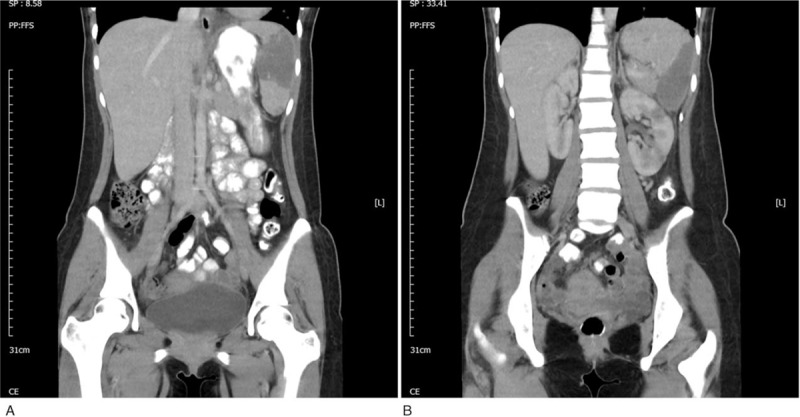
Abdominal computed tomography revealed splenic and left renal infarction.

An 18-year-old Indonesian woman came to Malaysia for a transnational medical consultation for intermittent low-grade fever, left leg weakness, and left abdominal pain for 3 weeks. She had medical history of mitral valve prolapse without mitral regurgitation. She presented with sudden onset of left leg weakness before fever. Intermittent low-grade fever, malaise, and poor appetite were noted after the leg weakness. She went to a clinic in her hometown, Indonesia, which diagnosed her with urinary tract infection and administered oral antibiotics. The fever subsided after the initiation of antibiotics. However, low-grade fever recurred after completing the antibiotics treatment. Left abdominal dull pain occurred. She was referred to Malaysia for further management. On examination, left leg weakness with muscle power of 3 was noted. Janeway lesions were found on her left palm and fingers. An apical pansystolic murmur was heard. The white blood cell count was 17.56 × 10^9^/L (normal, 4.0–11.3 × 10^9^/L) with 84% neutrophils (normal, 47%–80%); hemoglobin, 10.1 g/dL (normal, 12.5–16.0 g/dL); platelet count, 467 × 10^9^/L (normal, 140–450 × 10^9^/L); and erythrocyte sedimentation rate, 98 mml/hour (normal, 0–20 mml/hour). Three sets of blood culture revealed *S anginosus*, which was sensitive to all cephalosporin antibiotics. Transthoracic echocardiogram identified mitral valve prolapse with moderate eccentric mitral valve regurgitation with a 0.3 × 0.6-cm vegetation found on the left mitral valve. Brain magnetic resonance imaging showed disseminated septic emboli with microabscess in bioccipital and right posterior parietal juxtacortical region (Fig. 1). Abdominal computed tomography scan revealed splenic and left kidney infarction (Fig. 2). IE with splenic and left renal infarction and brain abscess were confirmed, and antibiotics treatment with penicillin was initiated. She fully recovered after 6 weeks of intravenous antibiotics treatment without any neurological sequelae. The patient and her family have provided informed consent for publication of the case.

## 3 Discussion

The prevalence of community-acquired IE in Asia is increasing.^[1]^*Staphylococcus* and *Streptococcus* spp. remained the most common microorganisms in IE. Most streptococci that cause IE are viridans streptococci. *S anginosus*, a member of the “*Streptococcus* milleri group” is often associated with invasive pyogenic infection, but not with IE.^[2–4]^ Most of the *S anginosus* are colonized in the oropharyngeal region, digestive system, and genitourinary system.^[5]^ The most prevalent underlying diseases associated with IE are intravenous drug addiction and valve heart disease. Young patients tend to have chronic rheumatic heart disease or congenital heart disease; however, elderly patients are likely to have degenerative valve disease.^[1]^ In our patient, fever started after the occurrence of gum bleeding. Oropharyngeal origin is highly suspected, and the patient had both invasive pyogenic infection in the brain and the less common infection IE with splenic and left renal embolization.

Mitral valve prolapse occurs in approximately 2% to 3% of the population in developed countries.^[6]^ Native valve disease, including mitral valve prolapse, bicuspid aortic valve, and previous IE, are the most common predisposing heart diseases associated with IE.^[7]^ However, mitral valve prolapse with moderate-to-severe mitral regurgitation has higher evidence of IE as compared with patients without mitral regurgitation.^[8]^ Although our patient had mitral valve prolapse, no mitral regurgitation was noted.

The diagnosis was made based on the modified Duke criteria. The keystone for the diagnosis of IE is the positive blood culture result. However, sometime blood culture result was negative because of the previous use of antibiotics. In previous studies, only one-third of all patients with IE had negative blood culture results.^[7]^

Transthoracic echocardiogram remains a good method to rapidly diagnose IE by searching for vegetation. However, the diagnostic rate in elderly patients who undergo transthoracic echocardiogram remains low.^[9,10]^

A vegetation of >1 cm may increase the incidence of embolization and mortality.^[11,12]^ Left-sided IE is always associated with splenic infarction and stroke.^[11]^ Surgical treatment is suggested because vegetation of >1 cm in left-sided IE is associated with higher mortality.^[13]^ In our case, vegetation was <1 cm and brain abscess remained small; thus, medical treatment was provided, and the patient responded well to the treatment. The muscle power of the left leg recovered after 6 weeks of antibiotics treatment.

IE should be highly suspected in young patients with underlying valve disease presenting with clinical symptoms. Oral hygiene in these patients remains an important factor to prevent IE. A study on mouthwashes showed reliable results on the importance of maintaining good oral hygiene^[14]^; however, further evidences are still required to prove whether it can be used to prevent IE.

## Acknowledgments

Authors thank everyone that contributed to this study.

## Author contributions

**Conceptualization:** Chiao-Wen Cheng, Chian Sem Chua.

**Supervision:** Cheng-Min Feng.

**Writing – original draft:** Chiao-Wen Cheng.

**Writing – review & editing:** Chian Sem Chua.
